# Lung ultrasound and mortality in a cardiogenic shock population: A prospective registry‐based analysis

**DOI:** 10.1002/ejhf.3692

**Published:** 2025-05-30

**Authors:** Guido Tavazzi, Costanza Natalia Julia Colombo, Matteo Pagnesi, Maurizio Bertaina, Andrea Montisci, Simone Frea, Marco Marini, Martina Briani, Lisa Patrini, Francesca Rossi, Letizia Bertoldi, Giulia Maj, Giovanna Viola, Carlotta Sorini Dini, Serafina Valente, Gaetano Maria De Ferrari, Nuccia Morici, Federico Pappalardo, Alice Sacco, on behalf of the Altshock Group

**Affiliations:** ^1^ Department of Clinical‐Surgical, Diagnostic and Pediatric Sciences University of Pavia Pavia Italy; ^2^ Intensive Care Fondazione Policlinico San Matteo Hospital IRCCS Pavia Italy; ^3^ PhD in Experimental Medicine, University of Pavia Pavia Italy; ^4^ Institute of Cardiology, ASST Spedali Civili di Brescia, Department of Medical and Surgical Specialties, Radiological Sciences, and Public Health University of Brescia Brescia Italy; ^5^ Division of Cardiology San Giovanni Bosco Hospital, ASL Città di Torino Turin Italy; ^6^ Division of Cardiothoracic Intensive Care ASST Spedali Civili Brescia Italy; ^7^ Division of Cardiology, Cardiovascular and Thoracic Department Città della Salute e della Scienza di Torino Turin Italy; ^8^ Division of Cardiology and ICCU, Department of Cardiovascular Sciences AOU delle Marche Ancona Italy; ^9^ Humanitas Research Hospital, IRCCS Rozzano Milan Italy; ^10^ Department of Anesthesia and Intensive Care Azienda Ospedaliera Universitaria SS. Antonio e Biagio e Cesare Arrigo Alessandria Italy; ^11^ Cardiology Department and De Gasperis Cardio Center ASST Grande Ospedale Metropolitano Niguarda Milan Italy; ^12^ Division of Cardiology, Department of Medical Biotechnologies University of Siena Siena Italy; ^13^ Department of Medical Sciences University of Torino Turin Italy; ^14^ IRCCS S. Maria Nascente‐Fondazione Don Carlo Gnocchi ONLUS Milan Italy; ^15^ Enna and Policlinico Centro Cuore G.B. Morgagni Kore University Catania Italy

**Keywords:** Lung ultrasound, Cardiogenic shock, B‐lines, Congestion, Mortality

## Abstract

**Aims:**

Lung ultrasound (LUS) is a widely used technique to assess de‐aeration in critically ill patients with respiratory failure. There is paucity of data on LUS in cardiogenic shock (CS). We sought to evaluate the epidemiology of lung congestion and its relation with outcome.

**Methods and results:**

The Altshock‐2 registry is a multicentre, prospective, observational registry including all‐comer CS patients. The LUS protocol included the examination of four zones using dichotomous assessment of lung congestion severity: ≤50% or >50%. LUS was performed at admission and at 24 h. Univariate and multivariate logistic regression analyses were performed. Overall, 185 patients (mean age 64.2 ± 13.5 years; 25.9% female) had a LUS at admission. A total of 128 patients (69.2%) had ≥50% of the investigated lung field with B‐lines. At univariate Cox regression analysis, B‐lines ≥50% at 24 h were significantly associated with increased 30‐day mortality (hazard ratio [HR] 4.705; 95% confidence interval [CI] 2.329–9.508) and the reduction of B‐lines during 24 h was associated with lower 30‐day mortality (HR 0.739; 95% CI 0.571–0.956; *p* = 0.021). Results were confirmed at multivariate analysis after adjustment for significant covariates: B‐lines ≥50% at 24 h (HR 2.23; 95% CI 1.042–8.654; *p* = 0.041) and the reduction in B‐lines from baseline to 24 h (HR 0.815; 95% CI 0.415–1.132; *p* = 0.039). The sensitivity analysis, excluding patients with cardiac arrest, led to significantly increased accuracy in outcome prediction.

**Conclusion:**

Assessment and monitoring of lung congestion with LUS over the first 24 h in patients with CS allow to further stratify clinical outcomes with higher accuracy when added to SCAI classification, especially when excluding patients with cardiac arrest at CS presentation.

## Introduction

Lung ultrasound (LUS) is widely validated to assess de‐aeration of the lung as identified by the presence and quantification of the so‐called B‐lines.^1^ The extent of lung field with B‐lines is associated with the severity of congestion and correlates in heart failure (HF) with pulmonary capillary wedge pressure (PCWP), either in preserved or reduced left ventricular ejection fraction, and with short‐ and long‐term outcomes.^2^, ^3^

Additionally, the number of B‐lines was also associated with the severity of clinical course in patients with acute myocardial infarction (AMI) leading to an improvement in the accuracy of prognostication when used in association with the Killip classification.^4^

B‐line quantification is usually reported according to different scores based on the sum of the number of B‐lines identified for each lung field, according to different zone protocols where the cutoff value for congestion severity varies according to the number of zones considered for each hemithorax.^5^ However, in case of severe congestion, it may be more challenging and time‐consuming to correctly number them, limiting its application in the acute setting (i.e. haemodynamic instability/cardiogenic shock [CS]). An eyeballing method to quantify the severity of lung de‐aeration (≤50% mild/moderate; >50% severe) has been suggested^6^ and validated in mixed respiratory failure populations^7^ showing the highest correlation with extravascular lung water.^8^ The prevalence of severe lung de‐aeration in patients with CS is well established, reported in up to 66% of cases contributing significantly to dismal prognosis.^9^

Despite the increasing and robust evidence of LUS usefulness in acute and chronic HF, there is a lack of prospective evaluation of B‐lines at admission, and their dynamic changes in relation to the clinical course and to the outcome in CS. We aimed to address this gap with a prospective cohort study based on the Altshock‐2 registry, a nationwide network tracking real‐time data on CS.

## Methods

### Study design

The Altshock‐2 registry (NCT04295252) is a multicentre, prospective, observational registry enrolling consecutive patients admitted for CS at 12 Italian centres since March 2020. CS was defined and stratified according to the Society for Cardiovascular Angiography and Interventions (SCAI) criteria.^10^ This study was approved by the Local Ethics Committee of Milano Area 3 of the ASST Grande Ospedale Metropolitano Niguarda (Milan) and then by all Local Ethics Committees of the hospitals participating in the registry. In accordance with the EU Regulation 536/2014, all competent patients provided written informed consent, whereas consent was waived for patients who were not competent on admission. The study was conducted in accordance with ethical principles based on the Declaration of Helsinki, International Conference on Harmonization for Good Clinical Practice, and the current ethical rules.^11^ The Strengthening the Reporting of Observational Studies in Epidemiology guidelines were followed for reporting study findings.^12^

Patient characteristics, in‐hospital data, in‐hospital and 30‐day outcome of all consecutively enrolled patients were collected and registered in an electronic case report form through the RedCap® platform. Laboratory, ultrasound and haemodynamic variables as well as SCAI shock stages, assigned according to the updated SCAI shock stage classification,^10^ were also reported at admission and at 24 h.

**Figure 1:**
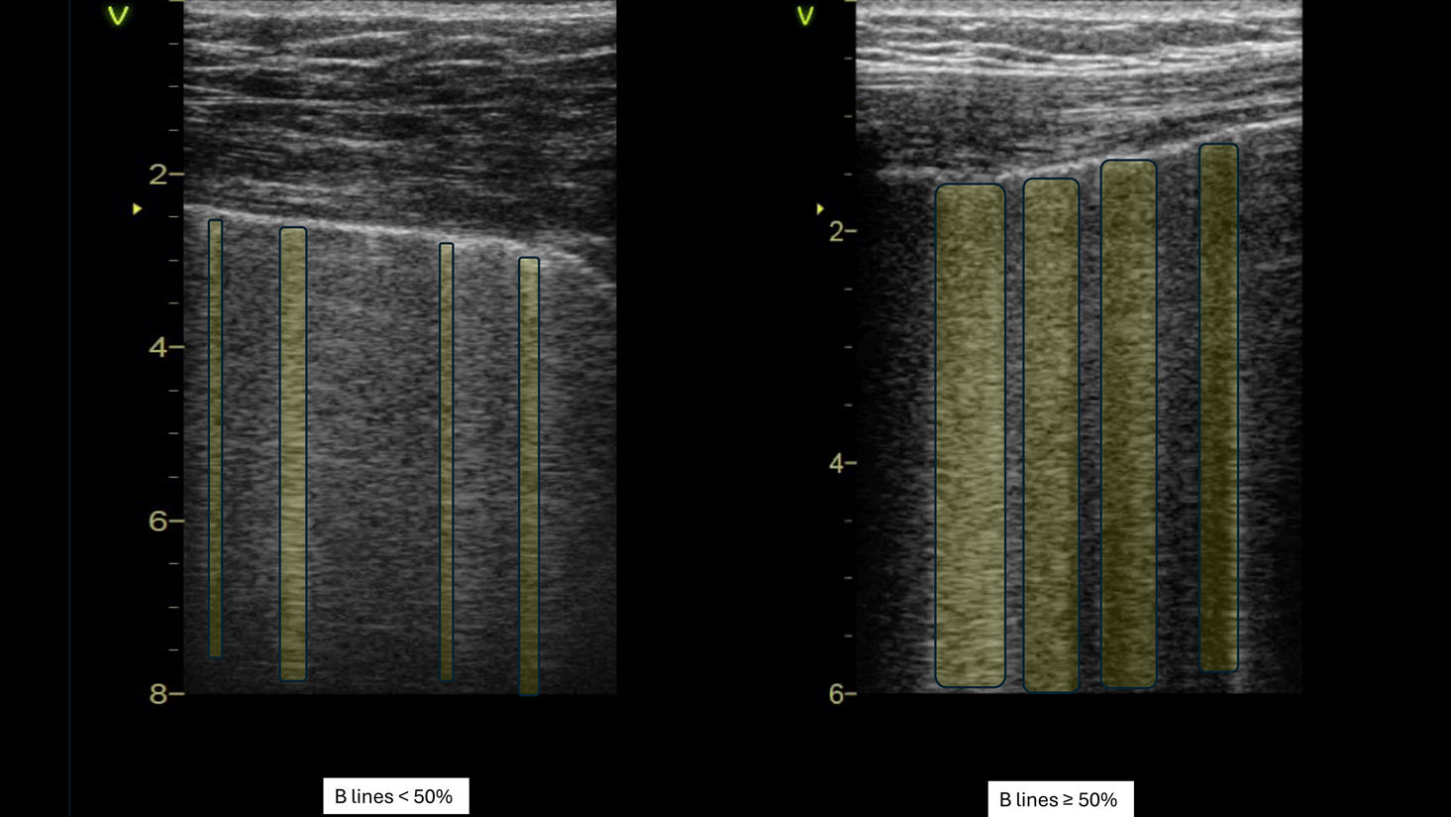
Lung ultrasound dichotomous evaluation. Example of B‐lines occupying <50% of the lung field (*left*) and B‐lines occupying ≥50% of the lung field.

The LUS protocol included the examination of four zones (one anterior and one posterior for pleural effusion assessment for each hemithorax) with transversal scanning using dichotomous assessment of lung congestion severity: ≤50% (mild/moderate) or >50% (severe)^6^ (*Figure* *1*). In case of heterogeneity of the extent of congestion, the results were averaged (one zone with ≤50% and three zones with >50% was considered >50% and vice versa). The zones with average worst congestion (≥50%) were considered.

To assess operator‐related bias and eventually improve the standardization of image interpretation, an intra‐ and inter‐observer variability test (including the number of B‐lines and dichotomic evaluation: <50% or ≥50%) was performed amongst physicians who performed LUS at the different enrolling sites and inter‐class correlation coefficient (ICC) was calculated (for B‐line counting and dichotomous evaluation).

The delta of B‐lines was considered as follows: group 1 with <50% both at baseline and 24 h; group 2 with ≥50% both at baseline and 24 h; group 3 with <50% at baseline and ≥50% at 24 h (worsening); and group 4 with ≥50% at baseline and <50% at 24 h (improving).

### Statistical analysis

Continuous variables are expressed as mean ± standard deviation or median and interquartile range, as appropriate, and were compared with the Student's *t*‐test and Mann–Whitney U test, respectively. Categorical variables are presented as numbers and percentages and were compared with the *χ* ^2^ or Fisher's exact test, as appropriate.

The primary endpoint was 30‐day mortality.

To evaluate the association between endpoints and ultrasound features, univariate and multivariate Cox regression analyses were performed, and hazard ratios (HRs) with the corresponding 95% confidence intervals (CIs) were calculated. Variables with a univariate value of *p* < 0.10 were incorporated into the stepwise selection, whereas age was forced into the multivariate analysis regardless of their association on the univariate analysis. A sensitivity analysis was then carried out excluding patients with cardiac arrest. A collinearity test was performed before entering the variables into the model. The receiver operating characteristic (ROC) curves were used to evaluate the discriminatory power of LUS at different time points. ROC analysis was also performed excluding cardiac arrest patients. ROC curve comparison was performed with the DeLong test.

To examine the intra‐ and inter‐operator agreement of the LUS evaluation, we applied the inter‐rater agreement (optimal agreement was defined by a *k*‐value >0.80). The ICC was used to measure the reliability of rating (strength of absolute agreement among the operators was considered poor, fair, moderate, strong, or almost perfect according to an ICC value <0.30, 0.30–0.49, 0.50–0.69, 0.70–0.89 and ≥0.90, respectively).^13^

Statistical significance was set at the two‐tailed 0.05 level. All the analyses were conducted with SPSS 29 (SPSS Inc., Chicago, IL, USA). The ROC curve comparison analysis was performed using MedCalc Statistical Software version 14.8.1 (MedCalc Software, Ostend, Belgium).

## Results

### Reproducibility

The reproducibility analysis was performed amongst all the clinicians who acquired the data by analysing 30 LUS exams as follows: number of B‐lines; lung field with B‐lines <50% or ≥ 50% for each clip. The set of exams was re‐evaluated by the same operator 2 weeks apart.

Absolute agreement was fair among the operators for the B‐line counting method (ICC 0.488; 95% CI 395–580) and was very strong when the dichotomous technique was applied (ICC 0.89; 95% CI 0.84–0.93).

### Population

Amongst 725 patients included in the registry, 185 (mean age 64.2 ± 13.5 years; 25.9% female) received a LUS at cardiac intensive care unit admission. LUS was performed at discretion of the physician without pre‐selected criteria. The comparison of the main patient characteristics between those who received LUS and those who did not are shown in online supplementary *Table Appendix* *S1*.

**Figure 2:**
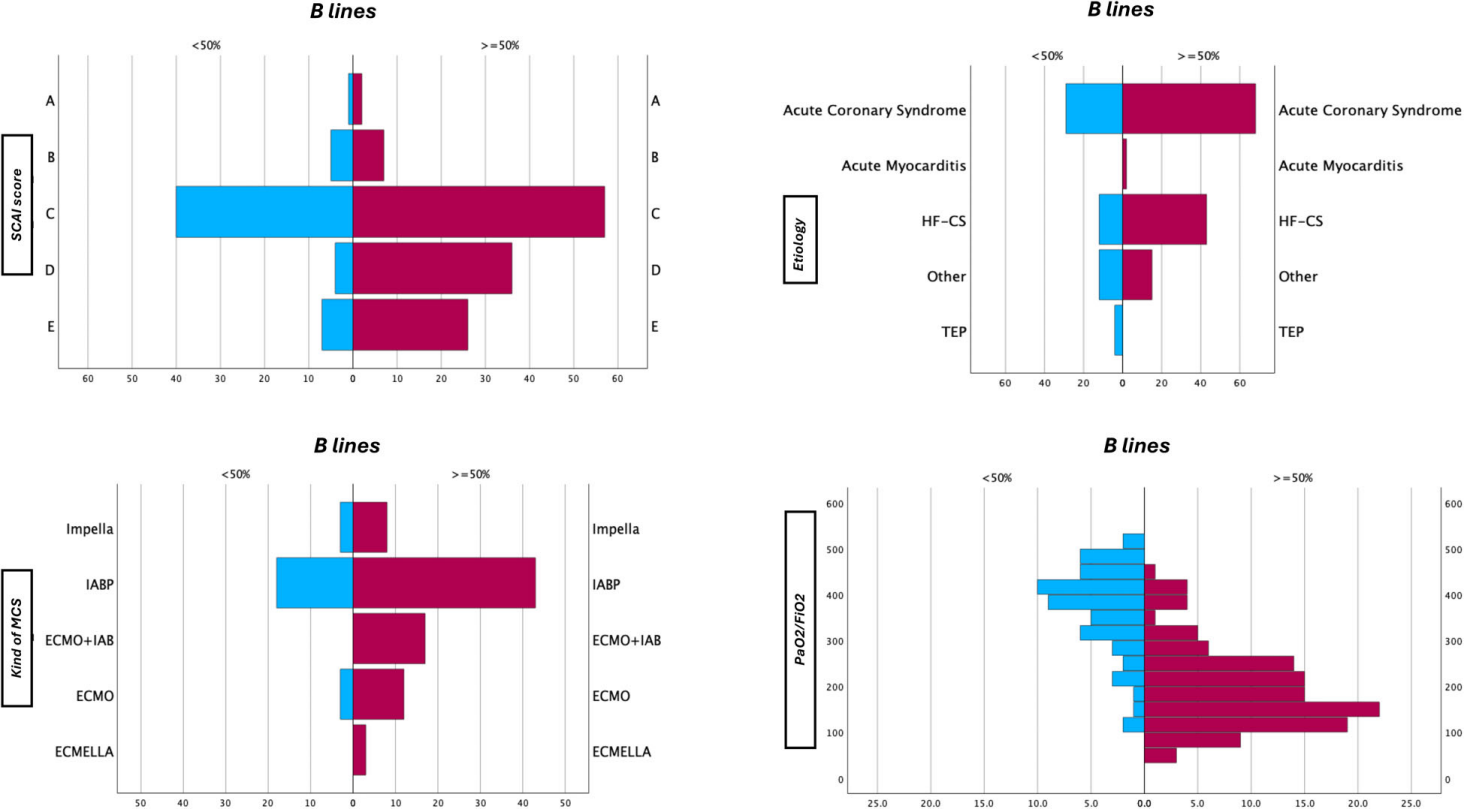
Population distribution characteristics. Population pyramid distribution for B‐lines at admission versus (*A*) Society for Cardiovascular Angiography and Interventions (SCAI) stage; (*B*) aetiology; (*C*) kind of mechanical circulatory support (MCS); arterial partial pressure of oxygen/fraction of inspired oxygen ratio (PaO_2_/FiO_2_). CS, cardiogenic shock; ECMO, extracorporeal membrane oxygenation; HF, heart failure; IAB, intra‐aortic balloon; IABP, intra‐aortic balloon pump; PTE: pulmonary thromboembolism.

Ninety‐seven patients (52.4%) had an AMI‐CS, 55 patients (29.7%) acute decompensated HF CS (ADHF‐CS), 17 patients (9.2%) presented with *de novo* HF, and 70 patients (37.8%) suffered from cardiac arrest. A total of 155 patients (83.8%) required respiratory support (34.1% non‐invasive ventilation and 64.3% invasive mechanical ventilation) and 108 patients (58.4%) received mechanical circulatory support (44.9% intra‐aortic balloon pump, 19.5% veno‐arterial extracorporeal membrane oxygenation [V‐A ECMO] and 8.1% axial flow devices). A total of 128 patients presented severe de‐aeration (≥50% B‐lines). *Figure* *2* shows the distribution of the study population according to SCAI stage and aetiology.

### Extent of pulmonary congestion

Characteristics of patients with <50% or ≥50% B‐lines on admission LUS are shown in *Table* *1*. Patients with ≥50% B‐lines at presentation had worse oxygenation and perfusion, identified as higher arterial lactates. Furthermore, patients with a higher degree of lung congestion (≥50% B‐lines) were more frequently treated with vasoactive pharmacological and mechanical circulatory supports (*p* = 0.04 and *p* = 0.008, respectively), whereas no differences were observed in the rate and kind of respiratory support (*Table* *1*).

**Table 1:** Differences according to B lines at presentation.

	**Total** ***n*** **=** **185**	**<50% B lines** ***n*** **=** **57**	**≥50% B lines** ***n*** **=** **12**	***p***
**Demographic**				
Female	48 (25.9%)	18 (37.5%)	30 (62.5%)	0.162
Age	64.25 ± 13.58	64.65 ± 14.86	64.1 ± 13.03	0.790
SOFA score	7.50 ± 5.12	5.59 ± 3.4	8.37 ± 5.61	< 0.001
SAPS II	46.76 ± 17.76	43.31 ± 16.87	48.31 ± 18	0.086
**Comorbidities**				
Hypertension	114 (61.5%)	36 (31.6%)	78 (68.4%)	0.279
Diabetes	54 (29.2%)	12 (27.8%)	39 (72.2%)	0.348
Lung disease	17(9.2%)	7 (41.2%)	10 (58.8%)	0.239
CKD	27 (14.6%)	8 (29.6%)	19 (70.4%)	0.682
Previous MI	57 (30.8%)	19 (33.3%)	38 (66.6%)	0.175
NYHA III‐IV	29 (15.7%)	11 (37.9%)	18 (62)	0.380
**Clinical presentation**				
Cardiac arrest	70 (37.8%)	21 (11.4%)	49 (26.5%)	0.852
SBP (mmHg)	101.13 ± 27.13	104.51 ± 23.41	99.60 ± 28.62	0.259
DBP (mmHg)	60.35 ± 17.22	63.32 ± 16.97	58.99 ± 17.23	0.116
MAP (mmHg)	73.98 ± 19.13	77.07 ± 17.98	72.58 ± 19.54	0.142
HR (bpm)	90.00 [73.00−108.00]	86.0 [75.0−106.5]	90.0 [72.8−110.0]	0.695
SaO_2_ (%)	96.07 ± 5.13	97.40 ± 2.90	95.47 ± 5.77	0.018
CVP (mmHg)	13.00 [9.00−16.00]	12.00 [7.50−17.50]	13.00[10.00−15.00]	0.862
pH	7.302 ± 0.166	7.354 ± 0.175	7.278 ± 0.156	0.004
PaO_2_ (mmHg)	122.08 ± 59.99	141.89 ± 66.96	113.00 ± 54.43	0.003
PaCO_2_ (mmHg)	38.85 ± 17.24	37.20 ± 19.32	39.62 ± 16.22	0.386
Lac (mmol/L)	4.64 ± 4.63	3.54 ± 4.17	5.13 ± 4.75	0.031
PaO_2_/FiO_2_ (mmHg)	250.78 ± 120.47	366.11 ± 96.38	196.05 ± 87.78	<0.001
SvO_2_ (%)	63.06 ± 12.42	60.27 ± 14.49	64.55 ± 10.99	0.105
Hb (g/dL)	12.86 ± 2.43	13.02 ± 2.06	12.79 ± 2.59	0.553
WBC (x109/L)	13.40 [9.70−18.25]	14.30 [10.40−17.95]	13.40 [9.50−18.55]	0.495
AST (U/L)	473.33 ± 996.31	303.17 ± 551.13	558.41 ± 1150.26	0.182
ALT (U/L)	392.85 ± 841.21	271.68 ± 504.85	446.23 ± 950.33	0.295
Bilirubin (mg/dL)	1.05 ± 0.97	1.15 ± 0.95	1.00 ± 0.97	0.364
Creatinine (mg/dL)	1.66 ± 1.35	1.57 ± 1.47	1.71 ± 1.30	0.508
GFR (mL/min/1.73 m^2^)	65.92 ± 38.00	70.55 ± 39.86	63.79 ± 37.09	0.271
Troponin I hs (ng/L)	63977.3 ± 217961.6	43778.0 ± 100350.5	72954.7 ± 253286.1	0.462
pro‐BNP (ng/L)	11771.63 ± 11095.57	15872.33 ± 9689.29	8491.07 ± 11354.60	0.086
BNP (pg/mL)	762.94 ± 1199.63	555.57 ± 896.29	834.13 ± 1285.58	0.339
LVEF (%)	25.01 ± 12.44	24.50 ± 11.89	25.27 ± 12.77	0.752
VIS	18.40 [6.13−35.00]	14.50 [4.45−37.00]	20.00 [10.00−35.00]	0.149
**Treatment**				
Vasoactive/inotropes	185	57 (30.8%)	128(69.2%)	0.040
Mechanical support	108 (58.4%)	25 (13.5%)	83 (44.9%)	0.008
IABP	83 (44.9%)	19 (33.3%)	64 (50.0%)	0.035
Impella	15 (8.1%)	3 (5.3%)	12 (9.4%)	0.344
V‐A ECMO	36 (19.5%)	4 (7.0%)	32 (25.0%)	0.004
Respiratory support	155 (83.8%)	43 (75.4%)	112 (87.5%)	0.400
NIV	63 (34.1%)	17 (29.8%)	46 (36.0%)	0.418
MV	119 (64.3%)	31 (54.4%)	88 (68.8%)	0.060
**Outcome**				
30‐days mortality	61 (33.0%)	13 (22.8%)	48 (37.5%)	0.05

Values are given as mean (standard deviation, or median [interquartile range], unless otherwise indicated.

Abbreviations: AST, aspartate aminotransferase; ALT, Alanine aminotransferase; BNP, B‐type natriuretic peptide; CRP, C‐reactive protein; CVP, central venous pressure; DBP, diastolic blood pressure; GFR, glomerular filtration rate; Hb, hemoglobin; HR, heart rate; IABP, intra‐aortic balloon pump; Lac, arterial lactate; LVEF, left ventricular ejection fraction; SBP, systolic blood pressure; MAP, mean arterial pressure, MCS, mechanical circulatory support; MV, mechanical ventilation; NIV, non‐invasive ventilation; PaO2, arterial partial pressure of oxygen; PaCO2, arterial partial pressure of carbon dioxide; PaO2/FiO2, arterial partial pressure of oxygen/fraction of inspired oxygen ratio; SaO2, arterial oxygen saturation; SvO2, venous oxygen saturation; VIS, vasoactive inotropic score; WBC, white blood cell; V‐A ECMO, veno‐arterial extracorporeal membrane oxygenation.

A total of 151 out of 185 patients had a LUS at 24 h and, according to the B‐line variation over the first 24 h, group 2 had a worse clinical and metabolic profile in terms of systolic blood pressure (*p* = 0.045), mean arterial pressure (*p* = 0.040), arterial partial pressure of carbon dioxide (*p* = 0.021), lactates (*p* < 0.001), arterial partial pressure of oxygen/fraction of inspired oxygen ratio (PaO_2_/FiO_2_) (*p* < 0.001) and liver enzymes (*p* < 0.001) (*Table* *2* and online supplementary *Table* *S2*).

**Table 2:** Clinical and biochemical profile divided into four groups according to the evolution of B‐line distribution between baseline and 24 h

	Group 1 (<50%)	Group 2 (stable ≥50%)	Group 3 (worsening)	Group 4 (improving)	*p*‐value
SBP, mmHg	110 (21.78)	102.84 (22.23)*	115.14 (14)	113.36 (18.48)	**0.045**
DBP, mmHg	63.3 (13.01)	58 (11.75)	62.61 (13.55)	62.91 (13.94)	0.189
MAP, mmHg	78.86 (13.9)	73 (11.77)*	80 (13.54)	79.74 (13.32)	0.40
HR, bpm	85.39 (20.44)	88.49 (21.65)*	82.36 (14.87)	81.68 (20.28)	0.374
SaO_2_, %	98.26 (2.08)	96.54 (5.18)	98.21 (1.71)	97.70 (1.94)	0.120
CVP, mmHg	9.65 (5.66)	10.60 (4.64)	8.86 (4.29)	9.91 (5.43)	0.823
pH	7.46 (0.05)	7.27 (0.88)	7.44 (0.05)	7.43 (0.47)	0.315
PaO_2_, mmHg	94.5 (27.18)	106.49 (50.64)	119.93 (52.43)	100.69 (28.12)	0.231
PaCO_2_, mmHg	33.86 (4.81)	41.94 (19.05)**	35.50 (4.43)	36.27 (5.87)	**0.021**
PaO_2_/FiO_2_	314.04 (67.03)	214.24 (96.28)**	266.57 (90.90)**	335.64 (141.58)	**<0.001**
Lactates, mmol/L	1.34 (0.42)	3.12 (2.98)**	1.47 (0.90)	1.54 (0.78)	**<0.001**
WBC, × 10^9^/L	12.63 (4.42)	14.93 (24.13)*	12.05 (3.57)	13.21 (5.72)	0.872
ALT, U/L	287.29 [185.34–687.54]	1088 [377.12–1861.23]**	110 [26.52–172.71]	440.96 [147.92–744.48]	0.134
Creatinine, mg/dl	1.26 (0.88)	1.83 (0.95)	1.44 (1.25)	1.51 (0.94)	0.77
GFR, ml/min/1.73 m^2^	77.71 (35.77)	59.39 (38.29)	87.85 (54.07)	75.19 (60.56)	0.152
CPR, mg/dl	11.59 [4.47–18.7]	13.9 [8.86–18.95]	8.9 [3.49–13.72]	12.96 [4–21.93]	0.624
TnI, ng/L	45 196 [7455–82 938]	184 294 [68030–300 558]	35 201 [11381–59 022]	106 667 [59551–153 783]	0.689
NT‐proBNP, pg/ml	7543 [1345–18 867]	15 329 [2709–21 949]	11 415 [9876–13 456]	11 239.29 [6187–19 658]	0.934
BNP, pg/ml	559 [183–934]	791 [514–1067]	557 [123–990]	639 [359–920]	0.757
LVEF, %	31.3 (15.2)	25.7 (13.2)	28.3 (6.75)	29.8 (12.44)	0.446

Values are given as mean (standard deviation), or median [interquartile range].

ALT, alanine aminotransferase; BNP, B‐type natriuretic peptide; CRP, C‐reactive protein; CVP, central venous pressure; DBP, diastolic blood pressure; GFR, glomerular filtration rate; HR, heart rate; LVEF, left ventricular ejection fraction; MAP, mean arterial pressure; NT‐proBNP, N‐terminal pro‐B‐type natriuretic peptide; PaCO_2_, arterial partial pressure of carbon dioxide; PaO_2_, arterial partial pressure of oxygen; PaO_2_/FiO_2_, arterial partial pressure of oxygen/fraction of inspired oxygen ratio; SaO_2_, arterial oxygen saturation; SBP, systolic blood pressure; TnI, troponin I; WBC, white blood cell.

*P*‐value between groups according to post‐hoc Bonferroni adjustment: *p < 0.01; **p < 0.001.

Twelve patients (6.5%) had a different B‐line distribution across the field investigated and needed the average. Two patients were initially considered having two zones with <50% and two zones ≥50% and an additional evaluation was required.

### Mortality

Overall 30‐day mortality was 33% (61 patients). Univariate and multivariate analyses are shown in *Table* *3*. Concerning the primary objective of the study, at univariate Cox regression analysis, B‐lines at baseline showed an odds ratio of 2.031 (95% CI 0.944–4.150; *p* = 0.052). The presence of ≥50% B‐lines at 24 h was significantly associated with increased 30‐day mortality (HR 4.705; 95% CI 2.329–9.508; *p* = 0.039). Conversely, the reduction of B‐lines over the first 24 h after admission was associated with a reduced 30‐day mortality (HR 0.739; 95% CI 0.571–0.956; *p* = 0.021).

**Table 3:** Regression analysis in all‐comer cardiogenic shock patients

	Univariate			Multivariate		
	HR	95% CI	*p*‐value	HR	95% CI	*p*‐value
B‐lines at baseline	1.878	0.908–3.886	0.089			
B‐lines at 24 h	4.705	2.329–9.508	0.039	2.23	1.0342–8.564	0.041
Δ B‐lines	0.739	0.571–0.956	0.021	0.815	0.415–1.132	0.039
SBP	0.987	0.976–998	0.019			
PaO_2_/FiO_2_	0.994	0.989–0.999	0.013			
Lactates	1.141	1.088–1.196	<0.001	1.115	1.028–1.208	0.008

Univariate and multivariate analysis after adjustment for age, cardiac arrest, SCAI stage, SOFA score, respiratory support and mechanical circulatory support.

CI, confidence interval; HR, hazard ratio; PaO_2_/FiO_2_, arterial partial pressure of oxygen/fraction of inspired oxygen ratio; SBP, systolic blood pressure; SCAI, Society for Cardiovascular Angiography and Interventions; SOFA, Sequential Organ Failure Assessment.

**Figure 3:**
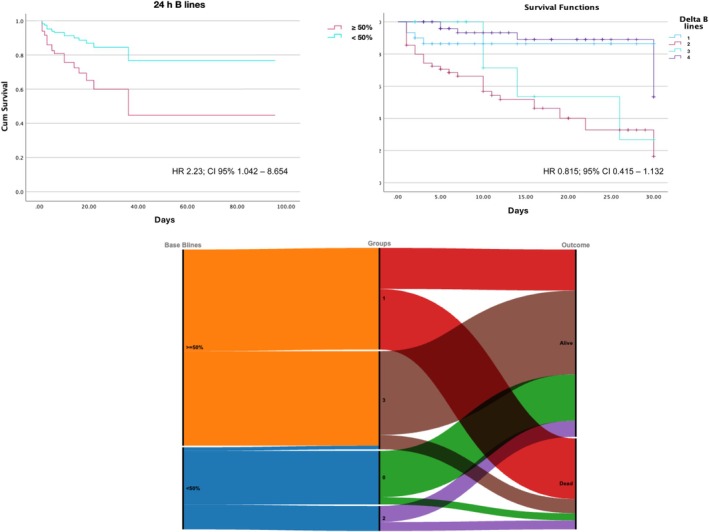
(*Upper panels*) Survival curves of B‐line extent (<50% or ≥50%) at 24 h (top left) and dynamic changes during the first 24 h after admission (top right) divided by group (group 1 with <50% B‐lines both at baseline and 24 h; group 2 with ≥50% B‐lines both at baseline and 24 h; group 3 with <50% B‐lines at baseline and ≥50% at 24 h [worsening]; group 4 with ≥50% B‐lines at baseline and <50% at 24 h [improving]). (*Lower panel*) Alluvial plot with three nodes: left y‐axis (baseline B‐lines); mid y‐axis (groups according to changes over the first 24 h) and right y‐axis (outcome). The graph should be read from left to right showing the flow between presentation, group allocation according to B‐line variation over the first 24 h and 30‐day outcome.

At multivariate analysis, also after adjustment for age, SCAI stage, Sequential Organ Failure Assessment (SOFA) score, respiratory and mechanical circulatory support, both retained in the model: ≥50% B‐lines at 24 h (HR 2.23; CI 95% 1.042–8.654; *p* = 0.041) and the reduction in B‐lines from baseline to 24 h (HR 0.815; 95% CI 0.415–1.132; *p* = 0.039) (*Table* *3* and *Figure* *3*). Lactates retained in the multivariate model.

At the sensitivity analysis, in univariate Cox regression analysis, B‐lines at baseline showed an OR 2.68 (95% CI 1.2932–7.254; *p* = 0.046). B‐lines ≥50% at 24 h were significantly associated with increased 30‐day mortality (HR 4.480; 95% CI 1.958–11.796). Conversely, the reduction of B‐lines over the first 24 h after admission was associated with a reduced 30‐day mortality (HR 0.561; 95% CI 0.234–1.098; *p* = 0.002).

At multivariate analysis, also after adjustment for age, SCAI stage, SOFA score, respiratory and mechanical circulatory support, ≥50% B‐lines at 24 h showed a HR of 6.480 (95% CI 2.658–15.796; *p* ≤ 0.001) and the reduction in B‐line from baseline to 24 h a HR of 0.345 (95% CI 0.201–3.124; *p* = 0.012).

**Figure 4:**
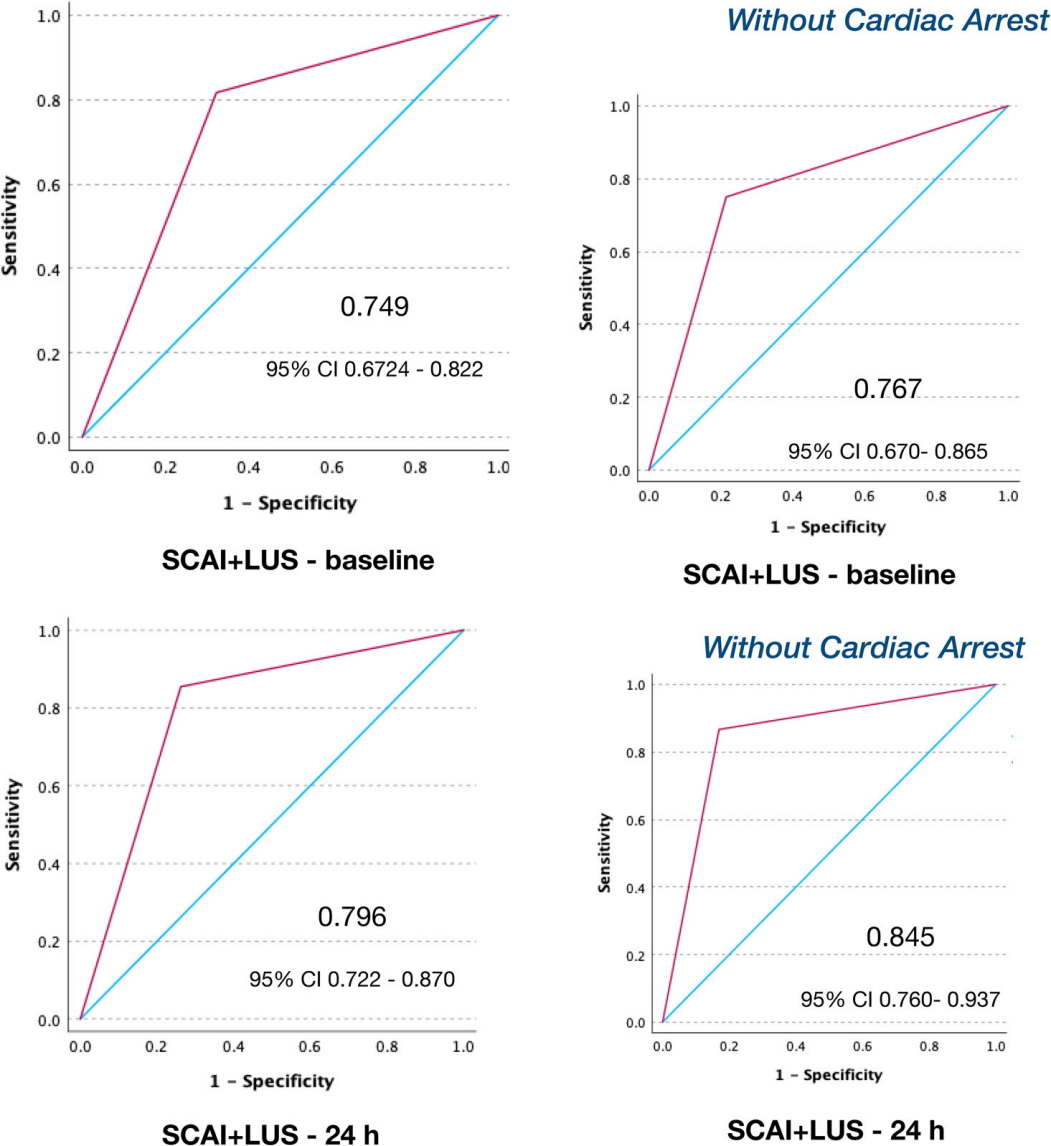
Receiver operator characteristic curves of Society for Cardiovascular Angiography and Interventions (SCAI) classification versus lung ultrasound (LUS) versus SCAI + LUS at baseline and 24 h in patients with and without cardiac arrest. CI, confidence interval.

Lung ultrasound and SCAI alone carried a moderate predictive values (online supplementary *Figure* *S1*). The addition of LUS increased the accuracy of the SCAI classification in predicting the outcome although without reaching statistical significance (*p* = 0.074), whereas it was significant when cardiac arrest patients were excluded (*p* = 0.023) as shown by the ROC curve in *Figure* *4*.

## Discussion

Although LUS has been widely used for patients with respiratory failure and HF, there are no reports on its relevance in assessing the extent of respiratory impairment and predicting outcomes in the CS population. In this multicentre, prospective, contemporary, real‐world registry including all‐comer patients with CS, the extent of B‐lines over the first 24 h was significantly associated with mortality (*Graphical Abstract*).

B‐lines represent the amount of de‐aeration which may result from cardiogenic interstitial oedema or inflammatory oedema in primary respiratory diseases. In the former, the distribution of B‐lines is homogeneous over the lung fields, whereas the latter is characterized by a patchy heterogeneous distribution.^1^ LUS has gained increasing attention in the cardiological population, although excluding mostly those presenting or developing CS.

An additional prognostic value over clinical evaluation (Killip class) was observed in AMI cohorts including a small sub‐population of CS patients.^4^, ^14^

Lung ultrasound was positively associated with increased natriuretic peptides in acute and chronic HF patients^15^, and a prospective study performed in an emergency department and a randomized controlled trial showed that LUS had higher accuracy as compared to standard imaging and peptides in diagnosing acute HF (area under the curve 0.95 vs. 0.87).

Moreover, multiple studies have demonstrated the accuracy of LUS in reflecting PCWP independently of ejection fraction.^3^ Specifically, more than six B‐lines for lung field correlate with PCWP >15 mmHg in HF with preserved ejection fraction and >20 mmHg in HF with reduced ejection fraction.^3^, ^16^

Additionally, B‐lines at discharge correlated with 60‐ and 180‐day rehospitalization and mortality. A systematic review on 13 studies on acute HF underlined the importance of B‐line monitoring, showing that in chronic and acute HF those with more severe congestion and persistence of B‐lines at discharge exhibited a high risk for HF rehospitalization or death.^17^

All previous studies focused on the scores, either on four or six zones, based on B‐line counting in haemodynamic stable patients.^18^ However, this method is time‐consuming, and its reliability has not been tested in the acute setting with haemodynamically unstable patients. The qualitative assessment of the de‐aeration severity scoring (<50% or ≥50%) was validated in critically ill patients with respiratory failure including, but not exclusively, patients with cardiogenic pulmonary oedema.^7^ Additionally, in a small sample of patients admitted to the intensive care unit due to acute respiratory distress syndrome, dichotomous evaluation showed the strongest association with extravascular lung water (*p* < 0.001, *r* ^2^ = 0.72) as compared with standard B‐line counting evaluation.^8^ We performed the first evaluation of such score exclusively in patients with cardiogenic pulmonary oedema, demonstrating higher ICC for the dichotomic approach (<50% or ≥50%) than for the B‐line counting technique. The application of this simplified scoring system allows distinguishing between severe and mild–moderate congestion, and it could be easily adopted in the acute setting, as CS, saving time but still providing prognostic stratification.

Notably, recent data have demonstrated that persistent congestion correlates with higher mortality, especially in ADHF‐CS patients, emphasizing the need for strict monitoring and timely therapeutic interventions.^19^ Accordingly, our group has recently demonstrated that the re‐classification of SCAI stage at 24 h was more accurate in stratifying outcome as compared to admission.^20^ Not surprisingly, the extent of congestion was associated with hypoxaemia. For the first time, a strong relationship between B‐line extent and signs of hypoperfusion, identified by increased lactate levels, was highlighted: this is probably explained by congestion leading to tissue hypoxia and therefore increased anaerobic metabolism.^21^ Indeed, B‐lines and lactates were the only other markers retained in the multivariable model. Lung failure is the most prevalent organ dysfunction in CS patients,^22^ and the rate of patients requiring mechanical ventilation is reported to be up to 66% of patients with AMI‐CS and 45% in ADHF‐CS, and respiratory failure has been repeatedly associated with mortality.^7^ In a substudy of the TRIUMPH trial on 260 patients requiring mechanical ventilation, each 1‐h delay from CS onset and invasive mechanical ventilation institution was related to a steep increase in mortality (OR 1.04; 95% CI 1.01–1.06; *p* < 0.001).^23^ Treatment of pulmonary congestion represents a critical aspect of CS management, just as restoration of cardiac output. In the recommendation of acute HF‐CS treatment, the application of cardiac ultrasound and LUS is regarded in the very early step as well as the application of positive pressure ventilation (PPV) in case of dyspnoea.^24^, ^25^ The PPV, besides improving oxygenation and preventing alveolar collapse, may promote reabsorption of oedema to the lymphatic system.^26^ Our results show the relevance of lung congestion as an expression of organ failure and how the assessment of lung congestion may help in improving outcome stratification since the first 24 h. We performed a sensitivity analysis excluding patients with cardiac arrest, according to the updated SCAI stratification, which highlights the importance of considering cardiac arrest as a modifier of outcome.^10^

Lung ultrasound congestion performed better when cardiac arrest patients were excluded likely for two reasons. Firstly, because resuscitated cardiac arrest populations are burdened by worse prognosis more commonly due to the multi‐organ failure anoxic injury.^27^ Secondly, early‐onset aspiration pneumonia occurs in up to 65% of cardiac arrest patients. At early stage, pneumonia may appear at LUS with signs of de‐oxygenation (B‐lines) rather than consolidation (tissue‐like). The presence of sub‐pleural consolidations may differentiate between B‐lines related to congestion and infection process,^28^ but their accuracy and onset have not been investigated in this specific setting.

Our results highlights the need for a multiparametric approach for a prompt identification of organ involvement in the CS pathophysiology for better phenotyping. If the implementation of targeted treatment aimed at reducing lung congestion and improving respiratory function, coupled with stricter monitoring through gas exchange and LUS, will demonstrate positive outcomes, it could represent a valuable addition to intensive care unit management protocols and it may suggest a redefinition of the SCAI parameters.

### Limitations

The main limitations of this study are related to the observational nature of the study. There is no control group as patients underwent LUS at total discretion of the physician. We provided a summary of the main characteristics of those receiving or not LUS in online supplementary *Appendix* *S1*. However, the selection bias may not be completely overcome by the methodological point of view.

The population was not completely homogeneous, however we adjusted the regression model also for the known variables potentially modifying the clinical outcome. In particular, the placement of V‐A ECMO and PPV could potentially represent a bias both in B‐line dynamic and oxygenation evaluation. Despite this potential bias, PaO_2_/FiO_2_ ratio was different between the two groups at baseline and remained different in group 4, and the B‐lines at 24 h and dynamic changes retained in the adjusted model.

Not all the physicians performing LUS were certified. However, all of them were long‐term practitioners using LUS currently in their clinical practice.

No sufficient invasive data were available to test the possible association between level of congestion and haemodynamics.

Although we consider the dichotomous evaluation feasible, easy, time‐sparing and, from our results, powerful in severity/outcome stratification, we do strongly acknowledge the need for validation in a prospective trial to properly compare this evaluation with the widely diffused B‐line counting system.

## Conclusion

Significant lung congestion is frequent in patients with CS, and dichotomic LUS evaluation is reliable to define the severity of congestion. Not only the extent of congestion itself but also its changes over the first 24 h is associated with short‐term outcome regardless of the CS underlying aetiology and severity as assessed by SCAI stages. Additionally, application of LUS to SCAI increased the accuracy in predicting the outcome, especially when excluding patients with cardiac arrest as CS presentation.

**Conflict of interest**: M.P. has received personal fees from Abbott Vascular, AstraZeneca, Boehringer Ingelheim, Novartis, Roche Diagnostics and Vifor Pharma. A.M. received speaker fees from Abiomed. M.M. has received personal fees from AstraZeneca, Boehringer Ingelheim, Bayer, Abiomed. All other authors have nothing to disclose.

## Supporting information


**Appendix S1.** Supporting Information.
