# Supplementary material for: Lung ultrasound and mortality in a cardiogenic shock population: A prospective registry‐based analysis
Source: Eur J Heart Fail. 2025 May 30;27(11):2594–603. doi: 10.1002/ejhf.3692 (PMC12765037; doi:10.1002/ejhf.3692)
Supplement: Supplementary file 1 — Appendix S1. Supporting Information. [file EJHF-27-2594-s001.zip › ejhf3692-sup-0001-Supinfo.docx]

Supplementary material

Table S1. Main characteristics between patients who received LUS and those who did not.

|  | **No LUS**  N= 540 | **LUS**  N= 185 | p value |
| --- | --- | --- | --- |
| Age | 64.6 ±16.6 | 63.84 ± 13.2 | 0.653 |
| Etiology |  |  |  |
| Ischemic | 67.5% | 32.5% | 0.223 |
| Non-Ischemic | 70.5% | 29.5% |  |
| Heart rate | 93.81 [76 – 110.9] | 93.57 [75.76 – 111.1] | 0.111 |
| SBP | 96.95 ±23.57 | 100.62 ± 26.29 | 0.528 |
| MAP | 70.55 ± 17.49 | 74.04 ± 18.65 | 0.019 |
| Lactates | 4.27 ± 4.04 | 4.63 ± 4.41 | 0.302 |
| CVP | 11.60 [7.82 – 13.65] | 12.74 [8.78 – 15] | 0.057 |
| pH | 7.36 ±0.12 | 7.33 ± 0.16 | 0.023 |
| PaO2/FiO2 | 272. 57 ± 121.72 | 252. 57 ± 121.72 | 0.092 |
| PaCO2 | 36.92 ± 13.61 | 38.50 ± 16.53 | 0.201 |
| Creatinine | 1.81 ±1.45 | 1.64 ± 1.28 | 0.163 |
| LVEF | 26.54 ± 12.68 | 26.21 ± 12.87 | 0.116 |
| V-A ECMO | 18.4% | 20.2% | 0.300 |
| IABP | 51% | 45% | 0.982 |
| Respiratory support | 68.8% | 33% | < 0.001 |
| Mortality | 34.6% | 31% | 0.130 |

*SBP, systolic blood pressure; MAP, mean arterial pressure; CVP, central venous pressure; PaCO2, arterial partial pressure of carbon dioxide; Lac, arterial lactate; PaO2/FiO2, arterial partial pressure of oxygen/fraction of inspired oxygen ratio; SvO2*

Table 2 supplementary. Comparison of hemodynamic and metabolic parameters between baseline and 24 h split by groups according to LUS changes

| Groups | SBP  t0 | SBP  24h | MAP  t 0 | MAP  24 h | pH  t0 | pH 24 | PaCO2  t0 | PaCO2 24 h | PaO2/FiO2  t0 | PaO2/FiO2  24h | Lactate  t0 | Lacate  24 h | CVP t0 | CVP 24 h |
| --- | --- | --- | --- | --- | --- | --- | --- | --- | --- | --- | --- | --- | --- | --- |
| 1 | 99  (±25) | 110 *  (±21) | 72  (±19) | 79  (±14) | 7.32 (±0.21) | 7.46* (±0.05) | 35  (±9) | 33  (±9) | 352  (±105) | 314  (±67)* | 3.85  (± 4.8) | 1.34§ (±0.42) | 13 (±6.8) | 10  (5.6)* |
| 2 | 98  (±31) | 103  (±22) | 72  (±21) | 78  (±14) | 7.25 (±0.16) | 7.27 (±0.83) | 38  (±15) | 42  (±19) | 209  (±98) | 214  (±96) | 6.32 (±5.57) | 3.12# (± 3) | 13 (±4) | 11  (±4) |
| 3 | 112  (±17) | 115  (±19) | 82  (±15) | 81  (±13) | 7.34 (±0.13) | 7.44 (±0.05) | 44  (±5) | 35  (±4) | 404  (±73) | 266^#^  (±90) | 3.23 (±4.07) | 1.47 §  (±0.90) | 12 (±5) | 9  (± 4) |
| 4 | 103  (±27) | 113  (±18) | 75 (±20) | 80  (±13) | 7.28 (±0.15) | 7.43^§^ (±0.04) | 42  (±6) | 36  (±5) | 198  (±87) | 266^§^  (±90) | 4.27 (±3.61) | 1.54 #  (±0.78) | 13 (±5) | 10  (±5) |

*P < 0.05

§ p < 0.01

# p < 0.001

Selection of variables to insert in the multivariable Cox regression model

The variable to input in the multivariable model were selected by choosing the clinically parameter which have been snown to be meaningful in previsou literature and which came out positive in the univariable model

Multicollinearity among variables was examined and co-linear variables were excluded by the model.

Figure 1 Supplementary material

Receiving operator curves of SCAI versus LUS at baseline and 24 hours
