# Supplementary figures and images for: Lung ultrasound and mortality in a cardiogenic shock population: A prospective registry‐based analysis
Source: Eur J Heart Fail. 2025 May 30;27(11):2594–603. doi: 10.1002/ejhf.3692 (PMC12765037; doi:10.1002/ejhf.3692)

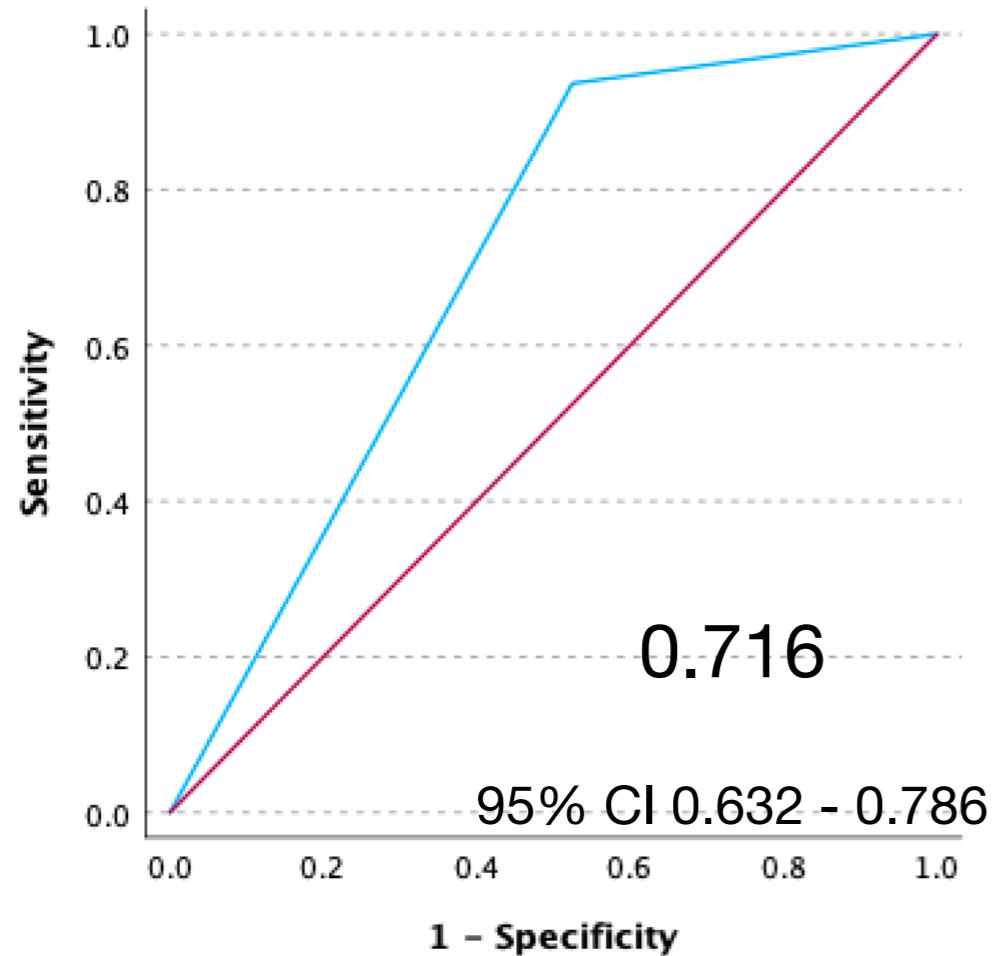

**LUS- baseline**

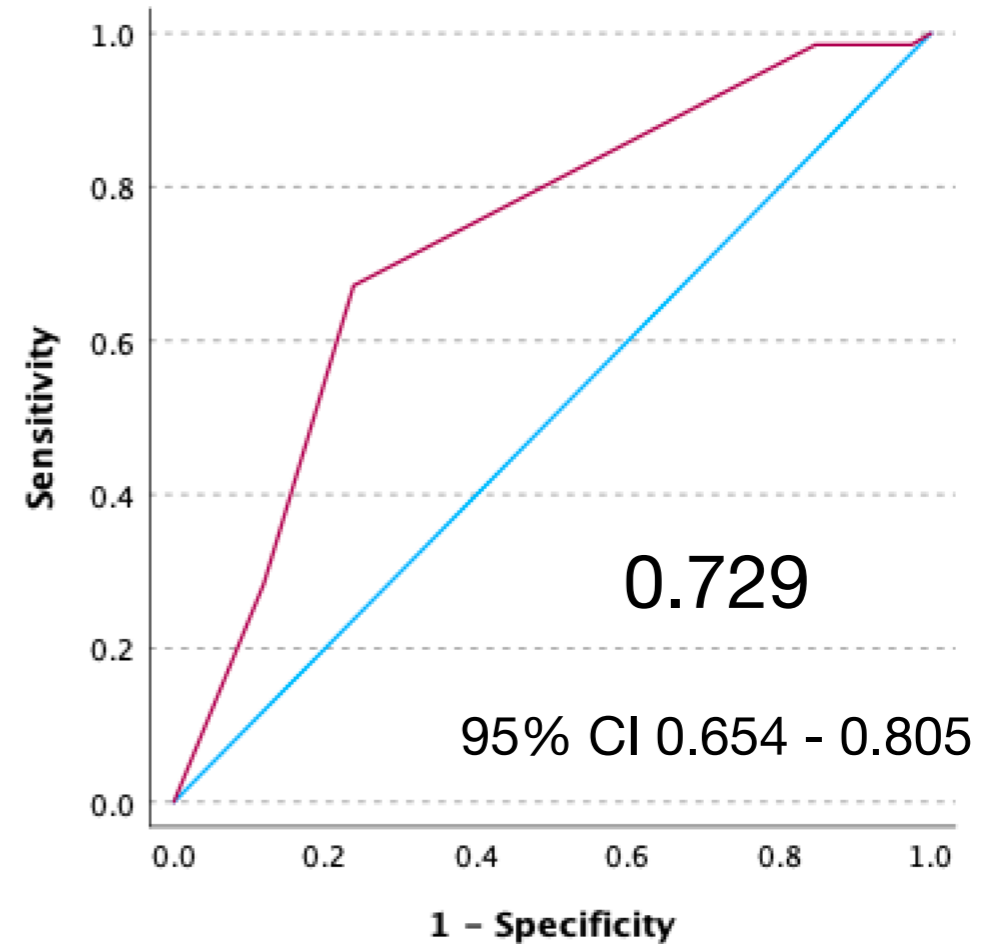

**SCAI - baseline**

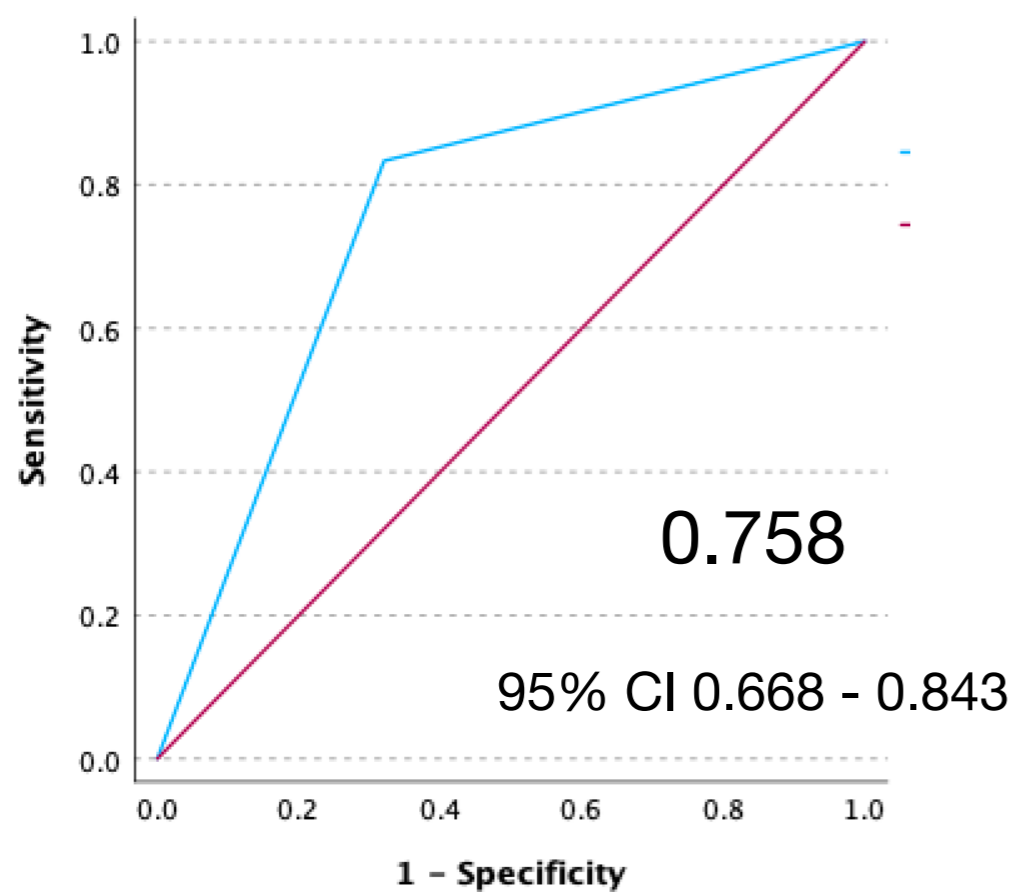

**LUS- 24 h**

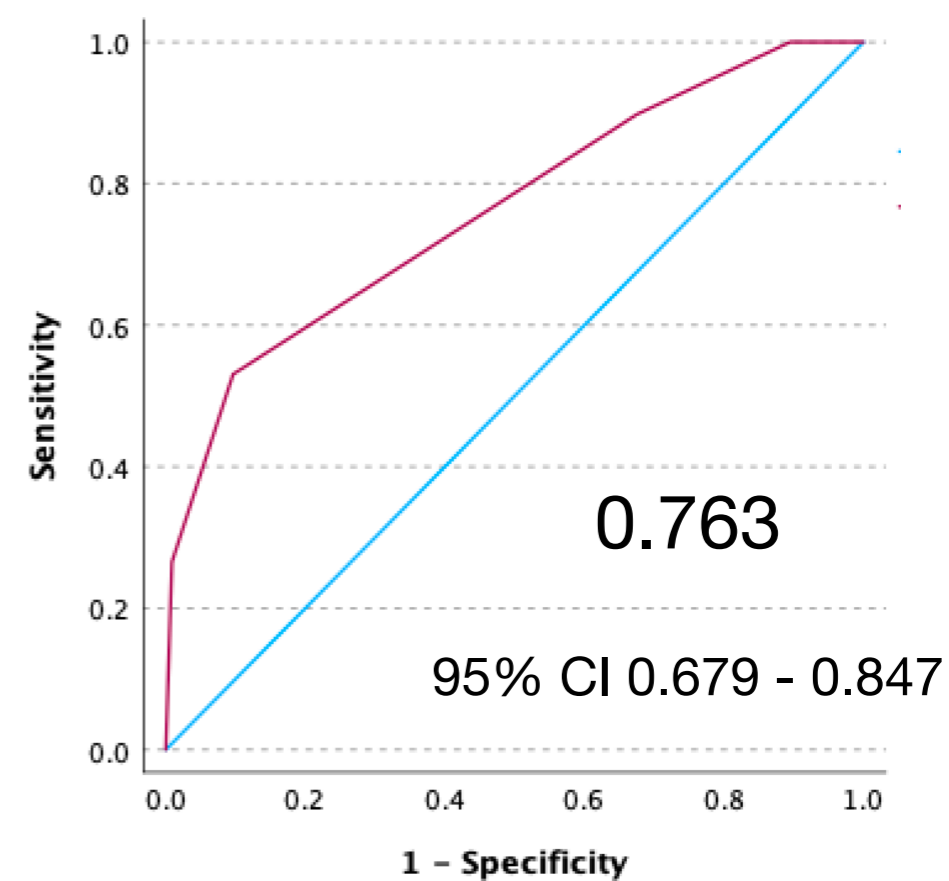

**SCAI - 24 h**

Supplement: Supplementary file 1 — Appendix S1. Supporting Information. [file EJHF-27-2594-s001.zip › ejhf3692-sup-0002-FigureS1.pdf]
